# Symptomatic Internal Carotid Agenesis in Children

**DOI:** 10.1155/2020/3290460

**Published:** 2020-04-25

**Authors:** Abdullah Alhaizaey, Ibrahim Alhelali, Musaed Alghamdi, Ahmed Azazy, Mohammed A. Samir

**Affiliations:** ^1^Division of Vascular Surgery, Aseer Central Hospital, Abha Maternity and Children Hospital, Abha, Saudi Arabia; ^2^Pediatric Intensive Care, Abha Maternity and Children Hospital, Abha, Saudi Arabia; ^3^Department of Pediatrics, Menoufia University, Menoufia, Shibin Al Kawm, Egypt; ^4^Aseer Central Hospital, Abha, Saudi Arabia

## Abstract

Carotid artery agenesis is a rare congenital anomaly, and there are controversies in the leading cause for it. We present a 6-year-old girl with resolved focal neurological ischemic stroke that showed bilateral internal carotid artery (ICA) agenesis. Through this paper, we highlight the carotid canal congenital obliteration hypothesis as it may be a risk for such finding.

## 1. Introduction

Agenesis of the internal carotid artery (ICA) is defined as the congenital absence of the internal carotid artery. It is rare, occurring in less than 0.01% of the population and is often an incidental finding [1, 2]. Bilateral ICA agenesis with patent common and external carotid arteries is extremely rare [3]. There is not yet an established hypothesis regarding the congenital and embryological anomalies that may lead to such agenesis. In this paper, we support the hypothesis that the embryological origin of the ICA differs from that of the common and external carotid arteries. The embryological development of the basal skull is important because the petrous bone carotid canal congenital diminution may lead to such anomalies [4]. Consent was taken for publishing such clinical finding.

## 2. Case Presentation

A six-year-old girl without any known illnesses was admitted to a pediatric hospital with sudden hemiplegia of the right upper and lower limbs. On clinical assessment, she was drowsy and her blood pressure was within the normal range, but she had sinus tachycardia (heart rate of 140 beats per minute) and her oxygen saturation was >96% in room air. She demonstrated total right-sided hemiplegia with power grade zero of four, loss of sensation with hypertonia, and exaggerated deep tendon reflexes.

**Figure 1:**
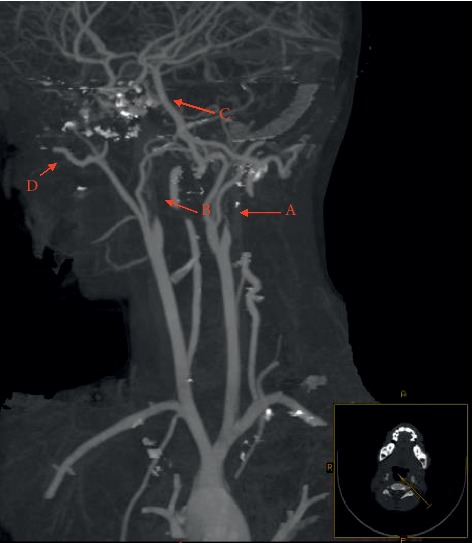
Sagittal view for CT head and neck with arterial phase contrast showed bilateral internal carotid artery (ICA) agenesis as shown by arrows A and B. Arrow C shows collaterals communicating with cerebral circulation that was supplied by an enlarged posterior communicating artery (POCMA) from a basilar artery (BA) and arrow D shows carotid rete mirabile from the external carotid artery.

**Figure 2:**
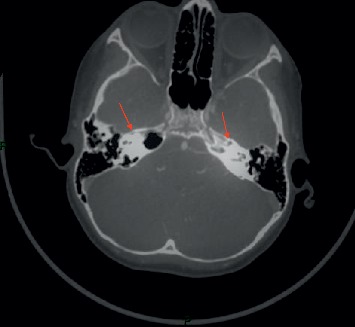
Skull base bone view with arrow showing obliteration for petrous bone carotid canal in the 6-year-old patient with bilateral ICA agenesis.

Family history was negative for similar conditions. The patient was delivered normally at 36 weeks of gestation; however, her mother had not followed prenatal and postnatal care advice. The patient was admitted and was resuscitated, which normalized her vital signs to 120/68 mm Hg for blood pressure and 90 heart beats/minute. Urgent radiological investigation including brain and neck computed tomography with and without arterial phase contrast was performed. A bilateral absence of the internal carotid arteries was seen with a complete bilateral obliteration of the petrous bone carotid canals. Multiple collaterals were seen communicating with the anterior cerebral circulation that was supplied by an enlarged posterior communicating artery (POCMA) from a basilar artery (BA) and carotid rete mirabile from the external carotid artery (Figures 1 and 2). Further radiological workup including magnetic resonance imaging with arterial (MRA) and venous (MRV) contrast enhancement of the brain and neck showed infarction of the left cortical area at the region of the central sulcus. No intracranial or extracranial aneurysms were seen, the flow to the superior sagittal sinus was normal, and there was no evidence of venous flow attenuation, thrombi, or venous occlusions. Echocardiography, hemoglobin electrophoresis, and a thrombophilia profile showed normal results.

The patient was started on 1 mg/kg of subcutaneous enoxaparin every 12 hours as an anticoagulant along with 5 mg/kg oral aspirin. She improved within 72 hours, and her weakness resolved completely, with a normal range of movement and sensation. She was discharged in good condition with oral aspirin. She has been followed up regularly for 6 months without any complaints.

## 3. Discussion

Agenesis of the ICA is rare. There is controversy regarding whether the common and external carotid arteries (CCA and ECA) have the same embryological origin as that of the ICA (3, 4, and 6). Few reported cases show bilateral agenesis of the ICA with patent ECA and CCA [4, 5] as in our case. This may support the hypothesis that the embryological origin of the ICA differs from that of the ECA and CCA. Carotid canal diminution or obliteration is usually associated with carotid artery agenesis rather than aplasia or hypoplasia. [6, 7] In our case, both ICA orifices were obliterated congenitally. Most reported cases were asymptomatic and were discovered incidentally during routine radiological examinations [4, 5], but a transient ischemic attack (TIA) causing a focal neurological lesion could occur. Therefore, carotid agenesis could be a differential diagnosis for TIA, especially in younger patients. Radiological examination using computed tomography with arterial and venous phase contrast or MRI and MRV for the brain and neck including the skull base may provide clues regarding the deferential diagnosis including such congenital agenesis, mainly focusing on basal skull canals as the occlusion of carotid canal may lead to obliteration of the internal carotid arteries [6]. In such cases, the brain blood supply is maintained through the collaterals; for example, in our patient, the posterior circulation enabled the blood supply.

## 4. Conclusion

ICA agenesis is considered a differential diagnosis for childhood ischemic brain stroke. ICA agenesis with a patent ECA and CCA may support the hypothesis of a separate embryological origin for the ICA. Bilateral agenesis highlights the importance of radiological review for skull base bone anatomy. Diminution or obliteration of the carotid canal increases the possibility that such agenesis is related to embryological skull base bony canal changes.
